# Intrapersonal strengths and interpersonal support: predicting academic buoyancy through psychological capital and growth mindset

**DOI:** 10.3389/fpsyg.2025.1584343

**Published:** 2025-07-17

**Authors:** Gui Xiao, Huifang Liu

**Affiliations:** Hunan Polytechnic of Environment and Biology, Hengyang, China

**Keywords:** academic buoyancy, psychological capital, social support, growth mindset, resilience, mixed-methods, Chinese university students

## Abstract

**Introduction:**

This mixed-methods study, situated within the framework of personality and social psychology, examines the interplay of psychological capital, social support, and growth mindset in predicting academic buoyancy among undergraduate English majors across three universities in mainland China.

**Methods:**

In the quantitative phase, established instruments were utilized to assess direct and mediated relationships among 516 undergraduate English majors, analyzed via structural equation modeling. The qualitative phase involved semi-structured interviews with 18 students to offer richer insights into these dynamics, analyzed using thematic analysis.

**Results:**

The quantitative analysis revealed that psychological capital and social support significantly and directly predict academic buoyancy (β = 0.413 and β = 0.341, respectively, *p* < 0.001). Furthermore, growth mindset was identified as a significant mediating variable in these associations (indirect effects: β = 0.098 and β = 0.126, *p* < 0.001). The proposed model accounted for 51% of the variance in academic buoyancy. Thematic analysis of qualitative data identified four core themes: navigating academic challenges, leveraging a growth mindset, the protective influence of social support, and the development of psychological capital.

**Discussion:**

Student narratives provided vivid accounts of overcoming academic obstacles, illustrating the mechanisms by which psychological capital and social support enhance adaptive beliefs and academic buoyancy. The findings underscore the importance of interventions designed to strengthen psychological capital, cultivate growth mindsets, and enhance social support networks within educational contexts.

## 1 Introduction

The demanding academic environment presents students with significant challenges that can impact their wellbeing, motivation, and overall success (Putwain et al., 2016). Effectively navigating these challenges—from daily setbacks like poor grades to tight deadlines and academic pressure—requires a crucial capacity known as academic buoyancy (Martin and Marsh, 2008). Crucially, academic buoyancy differs from the broader concept of resilience; while resilience typically refers to the ability to recover from major life adversities such as trauma or prolonged crises (Masten, 2001), academic buoyancy focuses specifically on students' capacity to successfully overcome routine academic setbacks and maintain their motivation and engagement in learning (Martin and Marsh, 2008). In the context of Chinese EFL university students, where intense academic competition and the complexities of mastering a foreign language are common, fostering academic buoyancy is particularly vital for sustaining engagement, mitigating stress, and ultimately achieving academic success (Fu, 2024; Mohammad Hosseini et al., 2024). Despite its importance, a comprehensive understanding of the psychological strengths and social resources that predict academic buoyancy, particularly within non-Western contexts, remains limited. This study addresses this gap by investigating how intrapersonal strengths, specifically psychological capital (PsyCap) and growth mindset, alongside interpersonal support, contribute to academic buoyancy among Chinese university students.

Psychological capital (PsyCap), a positive psychology construct, encompasses hope, self-efficacy, resilience, and optimism (Luthans et al., 2007). This study examines PsyCap as a whole, measuring all four components rather than focusing solely on resilience, to assess their combined influence on academic buoyancy. These resources drive goal pursuit and motivation (Luthans et al., 2004, 2010), enhancing academic achievement, engagement, and wellbeing (Carmona–Halty et al., 2019; Carmona-Halty et al., 2021; Martínez et al., 2019). Although resilience is a vital component of PsyCap, it operates alongside hope, self-efficacy, and optimism, which together provide a broader psychological foundation for managing daily academic demands. However, PsyCap's role in academic buoyancy, particularly alongside social and cognitive factors, needs further exploration. Positive psychology interventions cultivate such resources for flourishing (Lyubomirsky and Layous, 2013).

Social support, crucial for wellbeing and resilience (Cohen and Wills, 1985; Feeney and Collins, 2015), provides belonging, encouragement, and practical aid in academics, reducing stress and improving outcomes (Fu, 2024; Lei et al., 2022). It also fosters PsyCap components, such as self-efficacy and optimism, contributing to academic buoyancy (Fu, 2024). It enhances academic buoyancy and wellbeing through emotional and instrumental support (Bostwick et al., 2022; Granziera et al., 2022; Miller et al., 2013). Positive psychology underscores social connections for positive emotions and purpose (Ryff and Singer, 2008). However, the interplay between social support and PsyCap in fostering resilience requires clarification.

Growth mindset, the belief in ability development through effort (Dweck, 2006), promotes perseverance and a positive approach to challenges (Claro et al., 2016; Yeager and Dweck, 2012), positively impacting academic motivation, achievement, and wellbeing (Blackwell et al., 2007; Chan et al., 2022; Ortiz Alvarado et al., 2019; Zhao et al., 2024). Positive psychology highlights mindset's role in adaptive behaviors and growth (Peterson, 2000). Yet, growth mindset's mediating role between social/psychological resources and academic buoyancy, especially in non-Western contexts, warrants further investigation. Academic buoyancy, managing daily academic challenges, predicts motivation, self-concept, and performance (Collie et al., 2015; Datu and Yuen, 2018; Fu, 2024), differing sharply from resilience, which addresses major adversities such as prolonged crises (Martin and Marsh, 2008). Academic buoyancy reflects positive psychological functioning amidst academic stress (Keyes, 2002). Despite its importance, the combined influence of psychological and social resources and growth mindset's mediation in academic buoyancy is under-researched.

While existing research explores these constructs across cultures, the interplay of PsyCap, social support, and growth mindset in China's specific educational context remains under-examined. Integrated research on their combined effects and mechanisms on academic buoyancy is limited, hindering comprehensive understanding. This mixed-methods study addresses these gaps by investigating relationships between PsyCap, social support, growth mindset, and academic buoyancy among Chinese university students. It examines direct effects of PsyCap and social support on academic buoyancy and growth mindset's mediating role. Qualitative insights will enrich quantitative findings, providing deeper understanding of student experiences. By focusing on positive psychological resources, this study contributes to positive psychology's research on wellbeing and resilience. This study offers novel contributions by: (1) integratively analyzing PsyCap, social support, and growth mindset's combined influence on academic buoyancy; (2) extending academic buoyancy research to a non-Western, Chinese context; and (3) using mixed-methods to triangulate findings for a holistic understanding of academic resilience factors. Findings will inform targeted interventions and supportive learning environments, advancing both theory and practice in fostering student success, guided by positive psychology principles.

## 2 Literature review

### 2.1 Psychological capital

Psychological capital (PsyCap) has become increasingly important in understanding resilience, motivation, and success within academic settings. Defined as “an individual's positive psychological state of development” (Luthans et al., 2007, p. 3), PsyCap encompasses four core components: hope, self-efficacy, resilience, and optimism. Together, these elements function as psychological resources that empower individuals to overcome challenges and sustain performance across diverse contexts (Luthans et al., 2014; Newman et al., 2014). Hope involves the ability to set and pursue meaningful goals, even in the presence of obstacles, and relies on both willpower and waypower (Snyder, 2002). Self-efficacy, rooted in social cognitive theory, represents confidence in achieving desired outcomes through effort and persistence (Bandura, 1997; Luthans et al., 2007). Resilience provides the capacity to recover from setbacks and adapt to challenges, while optimism encourages individuals to view difficulties as opportunities for growth rather than insurmountable barriers (Masten, 2001; Seligman, 1998; Çavuş and Gökçen, 2015).

Although resilience is an integral part of psychological capital (PsyCap), it is not the sole focus; hope, optimism, and self-efficacy each contribute uniquely to academic buoyancy, collectively forming a robust psychological framework (Luthans and Youssef-Morgan, 2017; Ismail et al., 2024). Hope enables students to set goals and find ways to achieve them despite obstacles, helping them stay motivated after academic setbacks (Carmona–Halty et al., 2019). Recent research indicates that hope, as part of PsyCap, fosters persistence and enhances academic engagement, supporting students' ability to overcome challenges (Fu and Qiu, 2024). Optimism allows students to see difficulties as temporary and manageable, maintaining their effort and engagement in learning (Alsultan et al., 2023). Contemporary studies suggest that optimistic students, bolstered by PsyCap, cope more effectively with academic stress, reinforcing their academic buoyancy (Martínez et al., 2019). Self-efficacy, the belief in one's ability to succeed, drives students to tackle tasks with confidence and recover from failures (Lei et al., 2022). Recent evidence highlights that students with strong academic self-efficacy exhibit greater persistence and strategic adaptability, enhancing their capacity to bounce back academically (Guo et al., 2024). Together, these components interact synergistically—hope fuels motivation, optimism reframes setbacks, self-efficacy builds confidence, and resilience ensures recovery—strengthening students' capacity to handle daily academic challenges more effectively than resilience alone could (Zaeimzadeh and Jafari, 2023; Carmona-Halty et al., 2021).

Extensive research consistently highlights PsyCap's role in fostering positive academic outcomes, including enhanced engagement, improved wellbeing, and reduced stress, which collectively contribute to stronger academic performance (Carmona–Halty et al., 2019; Carmona-Halty et al., 2021; Fathi et al., 2023; Martínez et al., 2019; Vanno et al., 2014). Moreover, PsyCap is instrumental in fostering academic buoyancy, particularly in demanding educational settings (Fu and Qiu, 2024; Safriani and Muhid, 2022). For example, Safriani and Muhid (2022) found that PsyCap supported academic adjustment during the COVID-19 pandemic, enhancing adaptability under uncertainty. Likewise, Fu and Qiu (2024) showed that students with elevated PsyCap sustained engagement and reduced stress and burnout, with academic buoyancy acting as a moderator. These findings highlight PsyCap's capacity to mitigate academic challenges in high-pressure or blended learning environments. Recent studies also demonstrate PsyCap's role as a mediator and moderator in academic processes. Ismail et al. (2024) identified strong links between PsyCap and academic buoyancy, noting its multidimensional impact, including resilience, while Zaeimzadeh and Jafari (2023) connected PsyCap to enhanced critical thinking via positive learning experiences. Interventions designed to develop PsyCap, such as goal-setting, mastery experiences, and cognitive restructuring, effectively strengthen hope, self-efficacy, resilience, and optimism (Luthans et al., 2014; Luthans and Youssef-Morgan, 2017; Snyder, 2002), further promoting academic success and wellbeing.

In sum, PsyCap represents a robust set of internal resources critical for students' ability to thrive amidst academic challenges. While its direct link to academic buoyancy is established, understanding how cognitive factors like growth mindset and external supports such as social support might further enhance this relationship remains an important area for investigation, leading to our exploration of their interplay.

### 2.2 Academic buoyancy

Academic buoyancy has gained prominence in educational psychology as a construct that captures students' ability to manage everyday challenges and setbacks inherent in academic life (Martin and Marsh, 2008). Unlike resilience, which pertains to recovering from major life adversities such as prolonged trauma or significant disruptions (Martin and Marsh, 2009; Masten, 2001), academic buoyancy focuses narrowly on routine academic stressors—e.g., managing tight deadlines or rebounding from a low test score—emphasizing the capacity to recover from minor setbacks while maintaining motivation and persistence. This concept highlights the “ordinary magic” of everyday resilience, fostering a positive outlook and sustained engagement in learning (Alsultan et al., 2023; Martín, 2013). Academic buoyancy differs from both resilience and PsyCap in scope: resilience addresses severe adversities, while PsyCap encompasses a broader set of psychological resources—hope, self-efficacy, resilience, and optimism—that collectively support overall wellbeing and performance beyond just academic contexts (Luthans et al., 2007), whereas academic buoyancy targets students' responses to common academic challenges specifically (Martin and Marsh, 2008).

At the core of academic buoyancy is a dynamic interplay of cognitive and emotional processes that enable students to perceive challenges as manageable and adopt adaptive coping strategies. A key characteristic is a positive attributional style, where setbacks are attributed to external, unstable, and specific factors rather than internal, stable, and global ones, which helps students maintain hope and a sense of control (Peterson, 2000). Buoyant students often utilize strategies such as seeking help, adjusting learning methods, and employing positive self-talk to mitigate academic stress (Putwain et al., 2016, 2024). They are also skilled in emotional regulation, which prevents negative emotions like anxiety from interfering with their academic progress (Kritikou and Giovazolias, 2022). Furthermore, a growth mindset underpins buoyancy by fostering a belief in the ability to improve through effort and learning, encouraging students to view challenges as opportunities rather than threats (Dweck, 2006).

Research consistently demonstrates the positive outcomes associated with academic buoyancy, including increased motivation and engagement, a more positive academic self-concept, and greater confidence, all of which predict higher grades and academic persistence (Collie et al., 2015; Colmar et al., 2019; Datu and Yuen, 2018). Additionally, buoyant students report lower levels of stress, anxiety, and burnout, enhancing their overall academic wellbeing (Alsultan et al., 2023; Fu, 2024; Hoferichter et al., 2021). The reciprocal relationship between buoyancy and adversity further underscores its importance: successfully navigating challenges strengthens buoyancy, creating a feedback loop that enhances students' ability to handle future difficulties and fosters long-term resilience and academic achievement (Martin and Marsh, 2020). Beyond individual benefits, buoyant students also contribute to positive learning environments by promoting a sense of belonging and collective efficacy, which supports the development of a supportive classroom climate (Martin et al., 2013).

Recent studies have extended the relevance of academic buoyancy to diverse educational contexts, such as EFL learning (Derakhshan and Fathi, 2025; Mohammad Hosseini et al., 2024), doctoral education (Guo et al., 2024), and online learning settings (Xu and Wang, 2024). These findings highlight its universal applicability and its potential to address challenges across various student populations and learning environments. By fostering resilience, bolstering self-concept, and promoting adaptive strategies, academic buoyancy equips students to effectively navigate the demands of academic life. Further research should continue to explore its contributing factors and develop interventions that enhance this essential resource for student success. These efforts can provide actionable insights for educators aiming to cultivate supportive and empowering learning environments.

Given that academic buoyancy is influenced by internal cognitive processes and emotional regulation, exploring how a growth mindset (a key cognitive factor) might underpin and amplify this capacity is a logical next step. Furthermore, external social support is also recognized as crucial for navigating academic life, suggesting its potential to directly foster buoyancy or contribute to the psychological resources that enable it.

### 2.3 Growth mindset

Growth mindset, as conceptualized by Dweck (2006), is a foundational construct in educational psychology, emphasizing the belief that abilities are not fixed but can be developed through effort, dedication, and effective learning strategies. This perspective has transformed our understanding of learning, motivation, and achievement by encouraging individuals to view challenges as opportunities for growth and setbacks as part of the learning process (Blackwell et al., 2007; Yeager and Dweck, 2012, 2020). In contrast, a fixed mindset perceives abilities as innate and unchangeable, often leading to avoidance of challenges and fear of failure (Dweck, 2007). The distinction between these mindsets underscores the transformative impact of a growth mindset on students' approaches to learning and resilience (Burnette et al., 2023; Yeager and Dweck, 2012).

Students with a growth mindset demonstrate several adaptive beliefs and behaviors that support academic success. They recognize that intelligence and skills can be cultivated through learning and effort, viewing hard work and persistence as essential for achieving mastery (Blackwell et al., 2007; Claro et al., 2016; Fathi et al., 2024). This belief fosters a proactive approach to challenges, where setbacks are reframed as learning opportunities rather than failures (Yeager and Dweck, 2012). Furthermore, growth-oriented individuals often engage in positive self-talk, emphasizing their capacity for improvement and perseverance, which buffers against negative emotions and promotes wellbeing (Brooks et al., 2012). Research has also shown that a growth mindset can enhance resilience by encouraging students to view challenges as opportunities for growth and to persist in the face of adversity (Derakhshan and Fathi, 2024; Zeng et al., 2016).

The interplay between growth mindset and academic buoyancy is particularly significant, as it enables students to perceive challenges not as threats but as manageable tasks that contribute to personal development. This aligns closely with principles of resilience and self-efficacy (Luthans et al., 2014; Claro et al., 2016), as by fostering a sense of control and optimism, growth mindset enhances students' ability to recover from setbacks and persist in their academic endeavors (Putwain et al., 2016, 2024). This perspective also encourages help-seeking behaviors, such as collaborating with peers and seeking feedback, which are crucial for self-regulated learning and sustained engagement (Panadero, 2017; Pintrich, 2000).

Beyond its direct impact on academic buoyancy, growth mindset has been linked to broader positive educational outcomes, including improved academic performance (e.g., mathematics achievement, language learning) and enhanced psychological wellbeing (e.g., joy of learning, school connectedness; Chen et al., 2024; Chan et al., 2022; Valdez, 2024; Zeng et al., 2016). However, some research suggests that fixed mindsets may also have nuanced benefits, such as fostering a sense of educational purpose or perceived physical health, advocating for a more integrative perspective on mindset theories (Valdez, 2024).

The mediating role of academic buoyancy between growth mindset and other psychological constructs underscores its importance in resilience-building processes. For instance, Suharsono and Fatimah (2024) found that academic buoyancy mediates the relationship between growth mindset and psychological wellbeing, highlighting its role in translating motivational benefits into tangible outcomes. Similarly, Collie et al. (2015) identified a sense of control as a critical mechanism linking buoyancy and achievement, emphasizing the need to develop self-regulatory strategies alongside growth mindset principles.

The potential to cultivate growth mindset through targeted interventions further emphasizes its relevance to education. Programs designed to teach students about brain plasticity, provide opportunities for effort-based learning, and promote growth-oriented language have proven effective in fostering this mindset (Burnette et al., 2023; Yeager and Dweck, 2012). These interventions align with the foundational principles of growth mindset, encouraging students to embrace challenges, persist through difficulties, and maintain a lifelong orientation toward learning and development (Dweck, 2006).

Therefore, understanding growth mindset's role is crucial, not only as a direct contributor to academic buoyancy but also as a potential cognitive mechanism that might mediate the positive effects of internal strengths like PsyCap and external resources like social support on a student's ability to navigate daily academic setbacks. This positions growth mindset as a key intermediary in the holistic model of student resilience.

### 2.4 Social support

Social support, a foundational element of human resilience and wellbeing, refers to the interpersonal resources that enable individuals to cope with stress, overcome challenges, and thrive in various contexts (Cohen, 2004; Thoits, 1995). It is characterized by the perception of being cared for, valued, and connected, fostering a sense of belonging and security (Cobb, 1976). In academic settings, social support significantly contributes to students' ability to navigate the demands of learning, maintain motivation, and achieve their goals (Bostwick et al., 2022; Fu, 2024).

Social support takes multiple forms, including emotional, instrumental, informational, and appraisal support, each addressing different aspects of individual needs. Emotional support provides empathy and reassurance, helping individuals cope with emotional distress and maintain a positive outlook (Berkman et al., 1992). Instrumental support offers tangible aid, such as practical assistance or financial resources, alleviating stress by reducing the burden of challenges (House, 1981). Informational support, through advice and guidance, empowers individuals to solve problems and navigate stressors effectively (Thoits, 1995). Appraisal support enhances self-worth and perspective through feedback and affirmation, fostering a sense of competence and belonging (Cohen and Wills, 1985). These forms of support can originate from various sources, including family, peers, teachers, and mentors, with their quality and availability influenced by social networks and cultural contexts (Cohen and Wills, 1985).

The benefits of social support are extensive, consistently linked to improved psychological wellbeing, including greater happiness, life satisfaction, self-esteem, and resilience (Cohen, 2004; Siedlecki et al., 2014). Furthermore, social support buffers the adverse effects of stress, reducing symptoms of anxiety and depression while promoting a sense of control and optimism (Uchino et al., 1996). Its impact on physical health is equally profound, contributing to better cardiovascular health, faster recovery from illness, and increased longevity (Berkman et al., 1992).

Within educational contexts, social support plays a critical role in fostering academic buoyancy and enhancing PsyCap. It promotes resilience by providing encouragement and practical assistance, helping students recover from setbacks and persevere in their studies (Bostwick et al., 2022; Lei et al., 2022). Additionally, social support contributes to the development of PsyCap by fostering hope, self-efficacy, resilience, and optimism, thus enhancing students' confidence and capacity to succeed (Fu, 2024). Specifically, different types of social support nurture each PsyCap component in distinct ways. Emotional support from family or peers can boost optimism by offering reassurance and reducing stress, helping students stay positive during tough academic periods (Alsultan et al., 2023; Seligman, 1998). Instrumental support, like tutoring or shared resources, strengthens resilience by giving students tools to overcome obstacles, such as recovering from a poor grade (Granziera et al., 2022; Masten, 2001). Informational support, such as guidance from teachers, builds self-efficacy by providing strategies and knowledge that increase students' confidence in handling tasks (Bandura, 1997; Lei et al., 2021). Appraisal support, through encouragement and feedback from mentors, fosters hope by reinforcing students' belief that they can reach their goals (Luthans and Youssef-Morgan, 2017; Snyder, 2002). Together, these supports build PsyCap, which helps students manage daily academic challenges more effectively. Supportive relationships also facilitate a growth mindset by offering opportunities for constructive feedback and collaborative learning (Chan et al., 2022; Wentzel, 1998).

Recent research highlights the diverse effects of social support on academic outcomes. Social support moderates the link between self-efficacy and academic buoyancy, helping students transform confidence into resilience and better performance (Lei et al., 2022). It also mitigates academic stress, increasing engagement and reducing burnout (Af Ursin et al., 2021; Fu, 2024). These findings show how social support, academic buoyancy, and PsyCap interact, creating a cycle that bolsters student resilience and success. Longitudinal studies reinforce this dynamic, showing that strong school support leads to lasting gains in buoyancy, motivation, and engagement (Bostwick et al., 2022). Emotional and instrumental teacher support strongly promotes resilience and achievement, emphasizing the vital role of educators in fostering supportive settings (Granziera et al., 2022; Li et al., 2023). Peer support serves as a shield against academic challenges, enhancing buoyancy, easing test anxiety, and promoting wellbeing in high-pressure environments (Lei et al., 2021). Together, these studies underscore the multifaceted role of social support as a direct resource and moderator in resilience-building processes.

In summary, social support offers invaluable external resources that directly aid academic buoyancy and foster students' psychological capital. Its potential synergistic relationship with growth mindset in further enhancing academic buoyancy provides a compelling reason to explore its mediating role in our study's integrated framework.

### 2.5 The purpose of the study

This mixed-methods study investigates how PsyCap—a composite construct encompassing hope, self-efficacy, resilience, and optimism (Luthans et al., 2007)—along with social support and growth mindset, contributes to academic buoyancy among Chinese university students. The study examines PsyCap holistically, measuring all four components via the Chinese Psychological Capital Questionnaire (Ke et al., 2009), rather than isolating resilience, to understand their combined influence on students' ability to manage academic challenges. Driven by the need to understand how students can effectively navigate the challenges of higher education and thrive academically, this research draws upon a robust body of literature highlighting the significant role of psychological resources, social connections, and adaptive beliefs in promoting student success and wellbeing, consistent with the positive psychology emphasis on wellbeing and optimal functioning.

The quantitative phase of this study aims to examine the relationships between PsyCap, social support, growth mindset, and academic buoyancy. Specifically, it seeks to address the following research questions:

1. To what extent does PsyCap, encompassing hope, self-efficacy, resilience, and optimism (Luthans et al., 2007), along with social support, directly contribute to academic buoyancy in Chinese university students? This question is grounded in research demonstrating the positive impact of PsyCap (Luthans et al., 2007, 2010; Martínez et al., 2019) and social support (Cohen and Wills, 1985; Bostwick et al., 2022) on students' ability to cope with academic stressors, maintain motivation, and exhibit resilience.
2. **Does growth mindset mediate the relationships between PsyCap and academic buoyancy, and between social support and academic buoyancy?** This question explores the potential mediating role of growth mindset (Dweck, 2006) in explaining how PsyCap and social support influence academic buoyancy. It is informed by research suggesting that a growth mindset can foster resilience, persistence, and a positive approach to challenges (Claro et al., 2016; Yeager and Dweck, 2012), thereby contributing to greater academic buoyancy.

**Figure 1:**
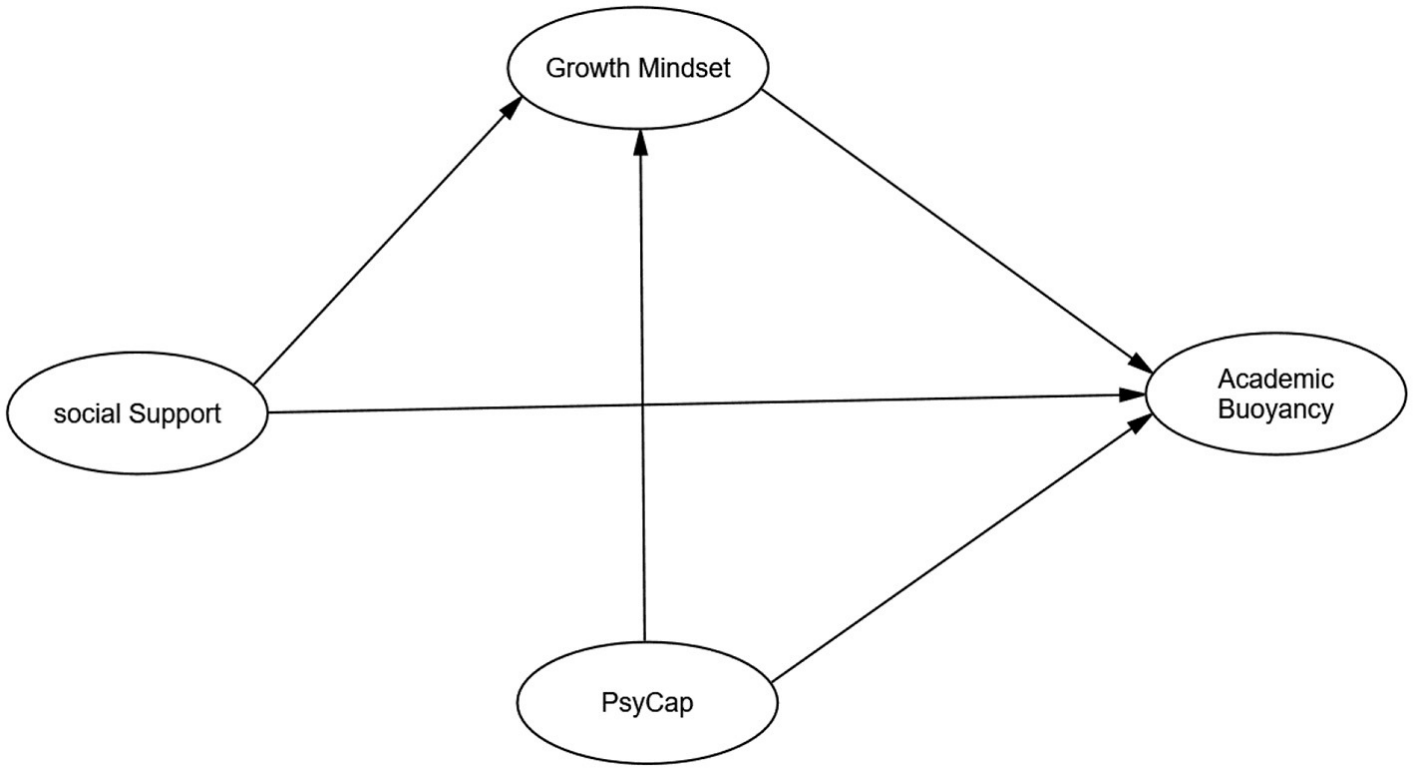
Conceptual framework illustrating hypothesized relationships between psychological capital, social support, growth mindset, and academic buoyancy.

By employing quantitative methods, including confirmatory factor analysis and structural equation modeling, this study seeks to provide empirical evidence for the hypothesized relationships between these variables and contribute to a deeper understanding of the factors that promote academic buoyancy in Chinese university students. The conceptual framework guiding these quantitative hypotheses is visually represented in Figure 1.

Complementing the quantitative inquiry, the qualitative phase of this study aims to provide a richer and more nuanced understanding of the lived experiences of Chinese university students in relation to academic buoyancy, PsyCap, social support, and growth mindset. Through semi-structured interviews, this study seeks to explore the following:

1. **How do Chinese university students perceive and experience academic buoyancy in their learning journeys?** This exploration will delve into students' own narratives of navigating challenges, bouncing back from setbacks, and maintaining motivation in their academic pursuits.
2. **How do students perceive the role of PsyCap, social support, and growth mindset in fostering their academic buoyancy?** This investigation will examine how students make sense of their psychological resources, social connections, and beliefs about ability in relation to their experiences of academic resilience and success.
3. **What specific strategies and resources do students draw upon to cultivate and maintain their academic buoyancy?** This inquiry will uncover the diverse ways in which students navigate challenges, seek support, and foster a positive mindset in their academic journeys.

## 3 Methods

### 3.1 Participants and procedures

This study employed a mixed-methods approach, beginning with a quantitative phase involving 516 undergraduate students majoring in English. Participants were recruited from three diverse universities in mainland China, ensuring representation across different institutional contexts and academic environments. These universities varied in terms of their location (urban vs. rural), size, and overall academic reputation, contributing to the generalizability of the findings. The participants were enrolled in a variety of English language programs, including literature, linguistics, translation studies, and English education, capturing a broad spectrum of specializations within the English major. Their ages ranged from 18 to 22 years (M = 20.1 years, SD = 1.2), reflecting the typical age range for undergraduate students in China. The sample consisted of 318 females (61.6%) and 198 males (38.4%), closely approximating the gender distribution observed in the broader population of English majors in Chinese universities. This balanced gender representation helps to mitigate potential bias and enhances the generalizability of the findings to both male and female students.

Participants for the quantitative phase were recruited using a convenience sampling method. Invitations to complete an online survey were distributed through university-wide email lists, student forums, and social media platforms targeting English majors. This multi-channel approach maximized participation and ensured a diverse sample within the target population. Participation was voluntary, and respondents were assured of strict confidentiality to promote honest responses. All data were anonymized and de-identified before analysis to safeguard participants' privacy.

Following quantitative data collection, a sub-sample of 18 students was purposefully selected for the qualitative phase. The selection criteria included academic performance (categorized as high, medium, or low based on the previous semester's GPA) and gender, ensuring an equal representation of male and female students. This purposive sampling strategy facilitated an in-depth exploration of students' diverse experiences with academic buoyancy. Semi-structured interviews were conducted via video conferencing, chosen for its flexibility and capacity to foster rapport while allowing participants to share their experiences in a comfortable setting.

Before participating in interviews, all students were informed about the study's purpose and procedures, and written consent was obtained from each participant. The study adhered to the ethical guidelines of the Declaration of Helsinki and received approval from the Ethics Committee of the University.

### 3.2 Data collection instruments

#### 3.2.1 Quantitative component

To quantitatively assess the key constructs under investigation, the following well-established and psychometrically sound instruments were employed:

##### 3.2.1.1 Psychological capital

The Chinese Psychological Capital Questionnaire (CPCQ; Ke et al., 2009) was used to assess participants' psychological capital. This 40-item instrument, specifically developed and validated for use with Chinese populations, includes two subscales: Task-Oriented Psychological Capital, measuring attributes such as self-confidence, optimism, resilience, and diligence (e.g., “I am confident that I can succeed at most things I try”), and Relationship-Oriented (Guanxi) Psychological Capital, assessing interpersonal qualities such as tolerance, respect, modesty, and gratitude (e.g., “I am willing to help others”). This instrument was chosen because it measures all four PsyCap components—hope, self-efficacy, resilience, and optimism—across its subscales, ensuring a full assessment of PsyCap rather than resilience alone. Participants responded on a 6-point Likert scale, ranging from 1 (strongly disagree) to 6 (strongly agree). A confirmatory factor analysis (CFA) verified the construct validity of this scale: χ^2^/df = 2.34, CFI = 0.93, TLI = 0.92, RMSEA = 0.05 (90% CI [0.04, 0.06]), and SRMR = 0.04. Cronbach's alpha (α) was 0.90, reflecting excellent reliability.

##### 3.2.1.2 Academic buoyancy

To measure academic buoyancy, a modified version of the Academic Buoyancy Scale (Martin and Marsh, 2008) was employed, adapting the original scale to address a wider range of academic challenges beyond mathematics. The scale consists of four items (e.g., “I bounce back quickly from setbacks in my schoolwork”) rated on a 7-point Likert scale from 1 (strongly disagree) to 7 (strongly agree). CFA results indicated a good model fit for this scale: χ^2^/df = 2.11, CFI = 0.94, TLI = 0.93, RMSEA = 0.04 [90% CI (0.03, 0.05)], and SRMR = 0.03. The scale demonstrated strong internal consistency, with Cronbach's alpha (α) calculated at 0.86.

##### 3.2.1.3 Social support

The Multidimensional Scale of Perceived Social Support (MSSS; Zimet et al., 1988) was utilized to evaluate participants' perceptions of social support from family, friends, and significant others (e.g., “My family really tries to help me”). This 12-item scale, which uses a 7-point Likert scale ranging from 1 (strongly disagree) to 7 (strongly agree), captures the multidimensional nature of social support. Construct validity was confirmed through CFA: χ^2^/df = 2.29, CFI = 0.92, TLI = 0.91, RMSEA = 0.05 [90% CI (0.04, 0.06)], and SRMR = 0.05. The scale's reliability was high, with a Cronbach's alpha (α) of 0.88.

##### 3.2.1.4 Growth mindset

The Growth Mindset Inventory (Dweck, 2006) was adapted and validated for the Chinese context through rigorous translation and back-translation procedures. This eight-item scale assesses beliefs regarding the malleability of intelligence and abilities (e.g., “You can learn new things, but you can't really change your basic level of talent”). Participants rated items on a 5-point Likert scale from 1 (strongly disagree) to 5 (strongly agree). CFA demonstrated acceptable fit: χ^2^/df = 2.18, CFI = 0.91, TLI = 0.90, RMSEA = 0.05 [90% CI (0.04, 0.06)], and SRMR = 0.04. The scale exhibited strong reliability, with a Cronbach's alpha (α) of 0.85.

#### 3.2.2 Qualitative component

##### 3.2.2.1 Semi-structured interviews

To gain a richer and more nuanced understanding of the quantitative findings, semi-structured interviews were conducted with the sub-sample of 18 purposefully selected students. This qualitative component aimed to explore students' lived experiences related to academic buoyancy, psychological capital, social support, and growth mindset in a more in-depth and contextualized manner. The interview protocol, developed based on the research questions and relevant literature, consisted of open-ended questions designed to encourage participants to elaborate on their perspectives, share personal anecdotes, and provide specific examples. Example interview questions include:

- Can you describe a time when you faced a significant academic challenge? How did you respond, and what strategies did you use to overcome it?”
- “How do you stay motivated and focused during difficult periods in your studies, and what strategies do you use to deal with setbacks or failures?”
- “Who do you typically turn to for support when you face academic challenges, and how does this support influence your ability to navigate those challenges?”
- “How do you perceive your academic abilities—do you believe they can be improved through effort? Can you share examples where you learned something new despite initial difficulty?”
- “When faced with academic challenges, how optimistic are you about overcoming them, and how do you view these challenges—as opportunities for growth or threats to your abilities?”

The interviews, conducted in Mandarin Chinese via video conferencing, provided a comfortable and convenient platform for participants to share their experiences. Each interview lasted ~45–60 min, providing ample time for in-depth exploration of the topics. All interviews were audio-recorded with the participants' explicit consent. The recordings were then transcribed verbatim and translated into English by a professional translator to facilitate analysis. This dual-language approach ensured accuracy and preserved the nuances of the participants' original responses.

### 3.3 Data analysis

To analyze the quantitative data, the study employed a two-step approach. First, confirmatory factor analysis (CFA) was conducted using AMOS 27.0 to assess the measurement model and ensure that the latent variables (psychological capital, social support, growth mindset, and academic buoyancy) were adequately represented by their respective observed indicators. Model fit was evaluated using several goodness-of-fit indices, including the chi-square statistic (χ2), the Comparative Fit Index (CFI), the Tucker-Lewis Index (TLI), the Root Mean Square Error of Approximation (RMSEA), and the Standardized Root Mean Square Residual (SRMR). Following the established guidelines (Hu and Bentler, 1999), acceptable model fit was indicated by a non-significant χ2 (or a χ2/df ratio < 3), CFI and TLI values greater than 0.90, and RMSEA and SRMR values less than 0.08.

Following the confirmation of the measurement model, structural equation modeling (SEM) was performed to test the hypothesized relationships among the variables. Maximum likelihood estimation was used to estimate the path coefficients, and bootstrapping (with 5,000 resamples) was employed to generate confidence intervals and assess the statistical significance of the indirect effects. The hypothesized model posited that psychological capital and social support would have both direct and indirect effects on academic buoyancy, with growth mindset serving as a mediator. To assess the mediating role of growth mindset, the procedures outlined by Preacher and Hayes (2008) were followed. Specifically, the indirect effects were examined by assessing the significance of the product of the path coefficients from the predictor to the mediator and from the mediator to the outcome variable.

To analyze the qualitative data, a thematic analysis approach was used, following the six-phase process outlined by Braun and Clarke (2006). This involved familiarizing ourselves with the interview transcripts, generating initial codes, identifying recurring themes, and refining these themes through an iterative process of review and discussion. Two independent researchers coded the data, and any discrepancies were resolved through consensus, ensuring the rigor and trustworthiness of the analysis. By integrating the qualitative findings with the quantitative results, a more comprehensive understanding of the factors influencing academic buoyancy was achieved. This mixed-methods approach allowed for triangulation of findings, enhancing the validity and credibility of the study's conclusions.

## 4 Results

### 4.1 Quantitative results

#### 4.1.1 Descriptive statistics and preliminary analyses

The final sample comprised 516 undergraduate English majors, with 318 females and 198 males, aged 18 to 22 years (*M* = 20.12, *SD* = 1.18). Data screening addressed missing values, which were minimal (< 3% per variable) and handled using expectation-maximization (EM). Multivariate outliers were assessed using Mahalanobis distance, with no cases exceeding the critical threshold (*p* < 0.001). Skewness and kurtosis values for all variables were within ±2, confirming approximately normal distributions.

Scale reliability, assessed via Cronbach's alpha (α), was strong for all constructs: psychological capital (α = 0.90), social support (α = 0.88), growth mindset (α = 0.85), and academic buoyancy (α = 0.86). These values indicate robust internal consistency for each instrument.

Table 1 presents descriptive statistics and bivariate correlations. Psychological capital exhibited the highest mean (*M* = 4.85, *SD* = 0.72), followed by academic buoyancy (*M* = 5.58, *SD* = 0.91), social support (*M* = 5.32, *SD* = 0.85), and growth mindset (*M* = 4.21, *SD* = 0.68). Correlation analysis revealed significant positive associations between all variable pairs (*p* < 0.001). Psychological capital showed notable positive correlations with social support (*r* = 0.62), growth mindset (*r* = 0.71), and academic buoyancy (*r* = 0.68). Social support also significantly correlated with growth mindset (*r* = 0.65) and academic buoyancy (*r* = 0.61). The strongest correlation was between growth mindset and academic buoyancy (*r* = 0.75). These correlations suggest a positive interconnectedness among psychological capital, social support, growth mindset, and academic buoyancy in this sample.

**Table 1:** Means, standard deviations, and correlations (*N* = 516).

**Variable**	**M**	**SD**	**1**	**2**	**3**	**4**
1. Psychological capital	4.85	0.72	—			
2. Social support	5.32	0.85	0.62^***^	—		
3. Growth mindset	4.21	0.68	0.71^***^	0.65^***^	—	
4. Academic buoyancy	5.58	0.91	0.68^***^	0.61^***^	0.75^***^	—

^***^p < 0.001 (two-tailed).

#### 4.1.2 Confirmatory factor analysis (CFA)

Confirmatory factor analysis (CFA) was performed in AMOS 27.0 to assess the hypothesized four-factor structure: psychological capital, social support, growth mindset, and academic buoyancy. Fit indices indicated a good model fit: χ^2^(731) = 1,245.87, *p* < 0.001, CFI = 0.92, TLI = 0.91, RMSEA = 0.05 [90% CI (0.05, 0.06)], SRMR = 0.06. These values suggest acceptable model fit (Hu and Bentler, 1999).

Standardized factor loadings ranged from 0.58 to 0.81, all significant at *p* < 0.001, supporting the distinctiveness of each construct. Items demonstrated meaningful and reliable loading onto their respective factors, aligning with theoretical expectations for psychological capital, social support, and academic buoyancy.

To further validate the four-factor model, two alternative models were tested. Model 1 combined growth mindset and psychological capital into one factor, yielding a poorer fit: χ^2^(734) = 1,685.21, *p* < 0.001, CFI = 0.88, TLI = 0.87, RMSEA = 0.07, SRMR = 0.08. Model 2 combined social support and growth mindset, also resulting in inadequate fit: χ^2^(736) = 1,872.34, *p* < 0.001, CFI = 0.85, TLI = 0.84, RMSEA = 0.08, SRMR = 0.09. Comparison of model fit indices and information criteria favored the original four-factor model. The four-factor model demonstrated superior fit and parsimony, with lower AIC (2,523.87) and BIC (2,585.03) values compared to the three-factor (AIC = 3,298.21; BIC = 3,356.11) and two-factor models (AIC = 3,487.34; BIC = 3,543.67). These results support the hypothesized four-factor structure as the most appropriate representation of the data.

#### 4.1.3 Structural equation modeling

**Figure 2:**
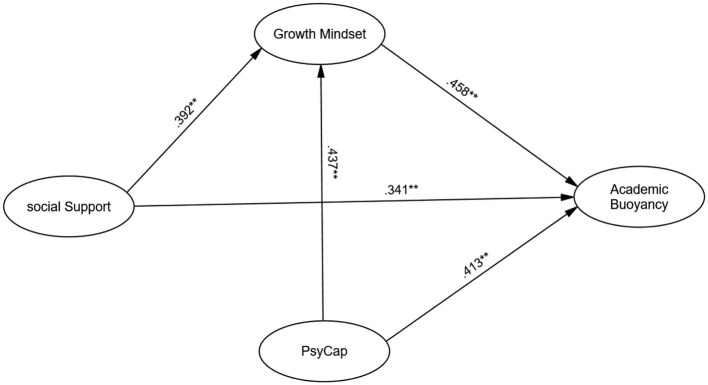
Structural equation model of the relationships among psychological capital, social support, growth mindset, and academic buoyancy. **p < 0.001.

Structural equation modeling (SEM) was conducted to test the hypothesized relationships. The model demonstrated good fit: χ^2^(731) = 1,245.87, *p* < 0.001, CFI = 0.92, TLI = 0.91, RMSEA = 0.05 [90% CI (0.05, 0.06)], SRMR = 0.06, meeting established criteria. The model accounted for 51% of variance in academic buoyancy, indicating substantial predictive power of the variables examined. The standardized path coefficients for this model are presented in Table 2 and visually summarized in Figure 2.

**Table 2:** Standardized path coefficients and confidence intervals for the SEM model (*N* = 516).

**Path**	**Effect**	**β**	**95% CI**	***p*-value**
Psychological capital → Academic buoyancy	Direct	0.413	[0.37, 0.46]	< 0.001
Social support → Academic buoyancy	Direct	0.341	[0.29, 0.39]	< 0.001
Psychological capital → Growth mindset	Direct	0.437	[0.39, 0.48]	< 0.001
Social support → Growth mindset	Direct	0.392	[0.34, 0.44]	< 0.001
Growth mindset → Academic buoyancy	Direct	0.458	[0.41, 0.51]	< 0.001
Psychological capital → Growth mindset → Academic buoyancy	Indirect	0.098	[0.07, 0.13]	< 0.001
Social support → Growth mindset → Academic buoyancy	Indirect	0.126	[0.09, 0.16]	< 0.001

All reported p-values are two-tailed. Bootstrap resampling = 5,000.

Standardized path coefficients (Table 2) revealed significant positive direct effects of psychological capital on academic buoyancy (β = 0.413, *p* < 0.001) and growth mindset (β = 0.437, *p* < 0.001). Social support also showed significant direct positive effects on academic buoyancy (β = 0.341, *p* < 0.001) and growth mindset (β = 0.392, *p* < 0.001). Growth mindset had the strongest direct effect on academic buoyancy (β = 0.458, *p* < 0.001).

The mediating effects of growth mindset were assessed using bootstrapped bias-corrected confidence intervals. The results confirmed that growth mindset partially mediated the relationships between psychological capital and academic buoyancy [β = 0.098, 95% CI (0.07, 0.13), *p* < 0.001] and between social support and academic buoyancy [β = 0.126, 95% CI (0.09, 0.16), p < 0.001]. While the indirect effects were smaller in magnitude than the direct effects, they underscore the crucial role of growth mindset as a mechanism through which both psychological resources and interpersonal support contribute to students' resilience.

To further validate the hypothesized model, an alternative model excluding growth mindset as a mediator was tested. This alternative model demonstrated a significantly poorer fit to the data, with χ^2^(df = 732) = 1,582.12, *p* < 0.001, CFI = 0.89, TLI = 0.88, RMSEA = 0.06, SRMR = 0.07, AIC = 3,196.12, and BIC = 3,258.28. In contrast, the hypothesized model achieved superior fit, with AIC = 2,523.87 and BIC = 2,585.03, indicating that it is more parsimonious and explanatory. The improved fit indices of the hypothesized model affirm the importance of growth mindset as a mediator in the relationships between psychological capital, social support, and academic buoyancy.

### 4.2 Qualitative results

The qualitative data, gathered through semi-structured interviews with 18 students, provided rich insights into the lived experiences of Chinese English majors and their perspectives on academic buoyancy, psychological capital, social support, and growth mindset. Thematic analysis revealed four overarching themes that aligned with and further illuminated the quantitative findings: (1) Addressing the Challenges of English Language Learning, (2) The Power of a Growth Mindset, (3) Supportive Relationships as a Buffer, and (4) Cultivating Psychological Strength.

#### 4.2.1 Addressing the challenges of English language learning

Students vividly described the multifaceted challenges inherent in learning English. These challenges extended beyond just linguistic difficulties, encompassing feelings of inadequacy, fear of judgment, and pressure to succeed. For instance, Wei (high-achieving female) confided, “Sometimes I feel so lost in my English literature class. The texts are so dense, and I struggle to understand the nuances. It makes me question my own abilities.” Similarly, Chen (low-achieving male) admitted, “I get really nervous before oral presentations. I'm afraid of making mistakes and being judged by my classmates. It's a constant source of anxiety.”

These anxieties and self-doubts often triggered the need for academic buoyancy. As Lin (medium-achieving male) explained, “When I get a low score on an English test, it can be really discouraging. I start to wonder if I'm good enough to continue in this major. But then I remind myself that everyone makes mistakes, and it's important to learn from them and keep trying.” This ability to bounce back from setbacks and maintain a positive outlook in the face of challenges was a common thread among the students, reinforcing the quantitative finding that psychological capital and social support were significant predictors of academic buoyancy.

Students employed various coping strategies to navigate these challenges. Many spoke about the importance of active learning and seeking support. For example, Li (high-achieving female) shared her proactive approach: “I'm not afraid to ask questions in class, even if they seem silly. I also try to find opportunities to practice speaking English outside of class, like joining the English club or chatting with international students.” This proactive engagement and willingness to seek help highlight the importance of a growth mindset and social support in fostering academic buoyancy, mirroring the quantitative findings.

#### 4.2.2 The power of a growth mindset

Students who demonstrated a growth mindset in their interviews viewed challenges not as obstacles but as opportunities for growth. They expressed a strong belief in their ability to improve their English skills through dedication and effort. Zhang (high-achieving male) articulated this perspective clearly: “I used to envy those classmates who seemed to pick up English effortlessly. But now I understand that language learning is a journey, and everyone progresses at their own pace. The key is to keep learning and growing.”

This growth mindset empowered students to embrace challenges and persevere through difficulties. Wang (medium-achieving female) shared an anecdote that illustrates this: “I used to struggle with writing essays in English. But instead of giving up, I sought feedback from my professor and practiced writing regularly. Gradually, I saw improvement, and now I actually enjoy writing.” This ability to view setbacks as learning experiences and maintain a belief in their potential for growth provides further evidence for the quantitative finding that growth mindset mediates the relationship between psychological capital and academic buoyancy.

#### 4.2.3 Supportive relationships as a buffer

Students consistently emphasized the importance of supportive relationships in their academic journeys. These relationships provided a sense of belonging, encouragement, and practical assistance, acting as a buffer against the stresses of language learning. For instance, Sun (medium-achieving male) described the unwavering support he receives from his family: “My parents have always encouraged me to pursue my passion for English, even though they don't speak the language themselves. They celebrate my successes and comfort me when I feel discouraged.”

Peer support also played a vital role. Zhao (low-achieving female) explained, “My classmates and I have formed a close-knit study group. We share resources, practice speaking together, and motivate each other to keep going. Knowing that I have their support makes a huge difference.” These narratives about the value of social support provide rich qualitative evidence to complement the quantitative finding that social support has a direct effect on academic buoyancy.

#### 4.2.4 Cultivating psychological strength

Students' narratives revealed a range of psychological resources that contributed to their academic buoyancy. They spoke about the importance of self-confidence, optimism, resilience, and hope. For example, Yang (high-achieving female) exuded confidence when she said, “I know that learning English can be challenging, but I'm determined to succeed. I believe in my ability to overcome obstacles and achieve my goals.”

This inner strength and resilience were evident in students' descriptions of how they handled setbacks. Wu (medium-achieving male) shared his approach: “When I face a setback, like failing a test, I try not to dwell on it. Instead, I analyze my mistakes, identify areas for improvement, and focus on moving forward.” These expressions of self-belief and resilience align with the quantitative finding that psychological capital has a strong direct effect on academic buoyancy.

Taken together, the qualitative findings provide rich context and deeper meaning to the quantitative results. The interviews revealed the specific ways in which psychological capital, social support, and growth mindset contribute to academic buoyancy in the lives of Chinese English majors. By giving voice to the students' experiences, the qualitative data elucidated the mechanisms underlying the quantitative findings, demonstrating how these variables interact in a dynamic and complex way to shape students' academic resilience. These findings underscore the importance of fostering not only students' cognitive abilities but also their psychological and social resources to promote academic success and wellbeing.

## 5 Discussion

This mixed-methods study investigated the interplay of PsyCap, social support, and growth mindset in fostering academic buoyancy among Chinese university students. The findings contribute valuable insights into the complex dynamics influencing students' ability to navigate higher education challenges and thrive academically, aligning with the core tenets of positive psychology which seeks to understand and promote optimal human functioning (Seligman and Csikszentmihalyi, 2000), while also raising questions for future research and practice.

Consistent with Hypothesis 1, PsyCap demonstrated a significant direct effect on academic buoyancy (β = 0.413, *p*<*0.0*01). This finding aligns with the understanding that PsyCap, as a composite construct of hope, self-efficacy, resilience, and optimism, collectively enables students to manage stress and setbacks effectively—not merely through resilience alone (Luthans et al., 2007, 2010; Carmona–Halty et al., 2019). To clarify, resilience within PsyCap supports recovery from adversity, but this study's outcome is academic buoyancy, which pertains to managing routine academic challenges. From a positive psychology perspective, cultivating these PsyCap components is crucial for enhancing individual strengths and promoting wellbeing (Luthans et al., 2004). These internal resources likely promote adaptive goal-setting, problem-solving, and perseverance, which are all vital for academic success. In the competitive Chinese higher education context, PsyCap proves particularly beneficial: hope fuels ambitious goals, self-efficacy builds confidence in academic tasks, resilience supports recovery from minor academic failures, and optimism encourages viewing challenges as growth opportunities rather than insurmountable barriers. Nevertheless, the moderate effect size suggests that other factors, such as cultural norms or peer interactions, also influence academic buoyancy (Kirikkanat and Soyer, 2018; Martínez et al., 2019).

Moreover, the study revealed a strong relationship between social support and PsyCap, suggesting that supportive relationships play a vital role in building students' psychological resources (Bostwick et al., 2022; Lei et al., 2022). Quantitative data showed a significant correlation between these variables (*r* = 0.62, *p* < 0.001), indicating that social support may enhance PsyCap components like self-efficacy and optimism (Alsultan et al., 2023; Carmona-Halty et al., 2021). For example, peer encouragement boosts academic confidence (Granziera et al., 2022; Lei et al., 2021), while family emotional support fosters hope and resilience during stress (Hoferichter et al., 2021). These findings align with research highlighting social support as a foundation for psychological strengths through reassurance and practical aid (Cohen and Wills, 1985; Feeney and Collins, 2015). Qualitative data reinforced this, with students reporting increased capability and hope from supportive networks (Carmona–Halty et al., 2019; Fu and Qiu, 2024). Social support directly aids academic buoyancy (β = 0.341, p < 0.001) and may amplify its effects by strengthening PsyCap (Af Ursin et al., 2021; Zaeimzadeh and Jafari, 2023). Future research could examine whether social support mediates or moderates PsyCap's influence on academic resilience.

Social support also exhibited a significant direct effect on academic buoyancy (β = 0.341, *p* < 0.001), supporting Hypothesis 2. This finding suggests that perceived support from family, peers, and teachers provides emotional reassurance, a sense of belonging, and practical assistance, which collectively bolster students' ability to cope with academic challenges (Bostwick et al., 2022; Hoferichter et al., 2021; Putwain et al., 2016). This underscores the positive psychology principle that strong social relationships are fundamental for wellbeing and resilience (Ryff and Singer, 2008). In the Chinese cultural context, where familial and peer connections are deeply valued, such support may serve as a critical buffer against academic stress, providing a safe haven for students to express their concerns and receive encouragement. However, the slightly smaller effect size compared to PsyCap might reflect cultural influences that discourage overt reliance on others. For example, Confucian values emphasizing independence and self-reliance may reduce students' willingness to fully leverage social support, potentially leading them to internalize struggles and downplay the importance of external assistance (Chan et al., 2022; Tang et al., 2019).

Growth mindset emerged as a partial mediator between PsyCap and academic buoyancy (β = 0.098, *p*<*0.0*01) and between social support and academic buoyancy (β = 0.126, *p*<*0.0*01), as hypothesized in Hypotheses 3 and 4. This finding underscores the role of growth mindset in translating psychological and social resources into academic buoyancy, as students who believe in the malleability of their abilities are more likely to view challenges as opportunities rather than threats (Claro et al., 2016; Yeager and Dweck, 2012). This aligns with positive psychology's emphasis on the power of cognitive reframing and adaptive belief systems in promoting positive outcomes (Peterson, 2000). For example, students with high PsyCap may leverage their optimism and self-efficacy to adopt growth-oriented perspectives, while those with strong social support might feel encouraged to persist through setbacks due to the belief that their efforts will ultimately lead to improvement. Growth mindset may also foster more effective self-regulation and coping strategies, as students with this mindset are more likely to seek feedback, analyze their mistakes, and adjust their learning strategies accordingly.

The partial nature of this mediation, however, invites deeper consideration. It indicates that while growth mindset is a significant pathway, it is not the *sole* mechanism through which PsyCap and social support influence academic buoyancy. Several factors might contribute to this partial effect. Firstly, individual differences in existing coping mechanisms or intrinsic motivation may allow some students to demonstrate academic buoyancy even without a highly developed growth mindset. Secondly, the efficacy of growth mindset interventions themselves can vary based on individual prior academic experiences or specific cultural norms regarding effort and ability (Chan et al., 2022; Macnamara and Burgoyne, 2023). Future research should therefore explore other potential mediators to gain a more comprehensive understanding. For example, self-regulation skills (Panadero, 2017; Pintrich, 2000) could serve as an additional mediator, where PsyCap and social support empower students to employ effective learning strategies, which in turn boosts buoyancy. Similarly, academic self-efficacy (Pajares, 1996) might be another key intermediary, as stronger beliefs in one's capability (potentially nurtured by PsyCap and social support) could directly lead to greater persistence and recovery from setbacks, independently of a generalized growth mindset. Investigating moderators such as prior academic achievement levels or specific academic stress types (e.g., test anxiety vs. project deadlines) could also further illuminate the conditions under which these relationships are strongest.

The qualitative findings enriched the quantitative results by revealing cultural nuances and contextual factors shaping students' experiences of PsyCap, social support, and growth mindset. For instance, some students expressed hesitancy in seeking help, reflecting concerns about “losing face” or burdening others. This reluctance to seek help, particularly from family and close friends, may be rooted in Confucian values that emphasize self-reliance and the importance of maintaining harmony within relationships (Chan et al., 2022; Zeng et al., 2016). These attitudes are consistent with Confucian values emphasizing self-reliance and familial harmony. Such cultural factors may limit students' willingness to fully utilize available support systems, potentially explaining the smaller effect of social support relative to PsyCap. Interventions promoting help-seeking behaviors in these contexts should consider these cultural sensitivities and emphasize the value of collaborative problem-solving and seeking guidance from trusted mentors without undermining students' sense of independence.

Another key insight was the coexistence of strong PsyCap and self-doubt among some students, highlighting the dynamic and context-dependent nature of resilience. Even students with high levels of hope, optimism, and self-efficacy may experience moments of fragility when faced with intense academic pressures or repeated failures, particularly in a high-stakes educational environment like China, where academic achievement is highly valued and competition is fierce. This nuanced understanding of resilience aligns with positive psychology's view of psychological strengths as dynamic and context-sensitive rather than fixed traits (Linley and Joseph, 2004). This aligns with theories suggesting that resilience is not a static trait but a fluctuating process influenced by situational stressors and feedback (Collie et al., 2015; Fu and Qiu, 2024). These findings suggest that resilience is not simply the absence of self-doubt but rather the ability to effectively manage and overcome these doubts, drawing on psychological resources and social support to maintain a positive trajectory. Positive psychology interventions often focus on building coping mechanisms to navigate such fluctuations in resilience (Seligman, 2011). These findings point to the importance of ongoing reinforcement through adaptive coping strategies, such as stress management training and supportive feedback, to help students sustain their resilience over time.

Overall, the findings of this study provide valuable insights into the psychological and social mechanisms underlying academic buoyancy in Chinese university students. From a positive psychology perspective, this study highlights the importance of fostering positive psychological resources and social supports to enhance student wellbeing and academic success. While PsyCap and social support emerged as significant predictors, the role of growth mindset as a partial mediator highlights the complex interplay of these factors. The study's mixed-methods approach offers a nuanced understanding of how resilience operates within cultural and contextual constraints. Future research should continue to explore alternative mediators and moderators to fully unravel the pathways to academic buoyancy, as well as develop culturally sensitive interventions to enhance students' academic resilience and wellbeing, drawing upon the principles of positive psychology to inform these efforts.

## 6 Implications

The qualitative findings provided valuable context, revealing how students leverage psychological resources, social networks, and adaptive beliefs to maintain resilience. These findings also highlighted the influence of cultural factors, such as the ambivalence toward help-seeking, on these relationships in the Chinese context. Together, these results contribute to a more nuanced understanding of how individual and social factors foster resilience in educational settings.

This study significantly contributes to the literature on academic buoyancy by incorporating PsyCap, social support, and growth mindset into an integrated theoretical framework. The findings underscore the multidimensional nature of resilience in academic settings, offering a nuanced understanding of how individual and social resources interact to foster students' ability to navigate challenges. By identifying growth mindset as a partial mediator, the research advances theoretical perspectives on the mechanisms through which PsyCap and social support influence academic buoyancy. Moreover, the study bridges a notable gap by applying the concept of PsyCap, originally explored in organizational psychology, to educational contexts, while also extending its relevance to non-Western cultural settings. This cross-cultural lens enriches the discourse on resilience by addressing contextual differences and emphasizing the global applicability of these constructs.

The practical implications of these findings provide actionable guidance for stakeholders in education, including educators, counselors, and policymakers. Enhancing PsyCap among students emerges as a critical focus, with interventions such as workshops aimed at fostering optimism, self-efficacy, and resilience. Techniques like role-playing or cognitive-behavioral approaches could be used to help students develop these psychological resources in a supportive and structured environment. Promoting growth mindset within educational frameworks further reinforces the importance of adaptive beliefs. Incorporating these principles into curricula and teaching practices, such as by offering constructive feedback that values effort and progress over innate ability, can help students reframe challenges as opportunities for development. Classroom environments that normalize and celebrate learning from failure could further strengthen these efforts.

In Chinese universities, PsyCap and growth mindset interventions could be implemented through practical programs suited to the local context. For example, universities could offer PsyCap development workshops where students practice goal-setting and pathway development to build hope, engage in mastery experiences through structured problem-solving activities to boost self-efficacy, and participate in cognitive restructuring exercises to cultivate optimism and resilience while reflecting cultural values like teamwork. These sessions could include activities like discussing past successes to further enhance optimism. Growth mindset could be explicitly introduced through teacher training, focusing on how to provide process-oriented feedback that highlights effort and strategy use over innate talent. This would involve adding short, interactive lessons within existing courses on the malleability of intelligence and abilities, using examples relevant to language learning. Peer mentoring programs could also be set up, where senior students guide younger ones, encouraging a belief in improvement through effort and shared learning. These steps would fit the Chinese emphasis on collective support and respect for education, making them both practical and effective.

The importance of social support networks also stands out as a key practical consideration. Educational institutions can enhance these networks by encouraging peer collaboration, mentoring programs, and initiatives that involve family engagement. For example, peer-to-peer support groups and resilience workshops led by faculty could provide students with both emotional encouragement and practical strategies for overcoming academic hurdles. The study also highlights the need to address cultural nuances in implementing these interventions. Particularly in contexts where help-seeking may be viewed as a sign of weakness, culturally sensitive approaches are essential. Anonymous support platforms and counseling services tailored to local cultural norms may reduce barriers and foster greater willingness to seek assistance.

## 7 Limitations

This study is not without limitations, which should be considered when interpreting the findings and planning future research. The cross-sectional design, while suitable for identifying relationships among PsyCap, social support, growth mindset, and academic buoyancy, restricts the ability to draw causal inferences. Longitudinal studies could provide more robust evidence by examining how these constructs interact over time, offering deeper insights into their dynamic influence on academic resilience. Another limitation lies in the sample characteristics, as the study focused exclusively on English majors in Chinese universities. While this specificity provided valuable insights into a particular academic and cultural context, it limits the generalizability of the findings to students in other disciplines or cultural settings. Future research should investigate whether similar relationships exist across a broader range of academic fields and populations outside China, which would enhance the applicability of the results.

A significant methodological limitation of this study stems from the exclusive reliance on self-report data, which introduces the potential for social desirability bias. Students might have answered questions in ways they thought looked good, especially in a culture that values modesty and harmony. For instance, they might have overstated their social support or downplayed struggles, potentially affecting the accuracy of the results. To mitigate this, future studies could incorporate alternative measures, such as teacher observations of student effort or academic performance data (e.g., grades), to provide a more comprehensive and triangulated picture. Combining interviews with these surveys, as was partially done in our mixed-methods approach, can also help capture a more authentic understanding of students' experiences, particularly in a Chinese setting where direct reporting can be nuanced.

The study also considered growth mindset as a mediator but did not examine other potential mediators or moderators that could influence the observed relationships. Constructs such as self-regulation skills and intrinsic motivation might offer additional explanations for how PsyCap and social support contribute to academic buoyancy. Similarly, moderators like socioeconomic status or academic self-concept could shed light on factors that enhance or diminish the strength of these relationships. Expanding the model to include these variables would allow for a more comprehensive understanding of academic buoyancy.

Finally, despite careful adaptation and validation of the measurement tools, cultural nuances in how PsyCap and growth mindset are conceptualized and experienced may have influenced the findings. Although the instruments were tailored to the Chinese context, subtle differences in interpretation could affect the validity of the constructs. Future research could benefit from employing mixed-methods designs to refine these constructs further and ensure their cultural appropriateness in diverse settings. By addressing these limitations, future studies can build on the current findings to advance theoretical and practical understanding of academic buoyancy across various contexts.

## Data Availability

The datasets generated and analyzed during the current study will be made available from the corresponding author upon reasonable request. Data will be shared in anonymized form, consistent with ethical and data privacy policies. Requests to access these datasets should be directed to the corresponding author.
