# Supplementary material for: Intrapersonal strengths and interpersonal support: predicting academic buoyancy through psychological capital and growth mindset
Source: Front Psychol. 2025 Jul 17;16:1584343. doi: 10.3389/fpsyg.2025.1584343 (PMC12311807; doi:10.3389/fpsyg.2025.1584343)
Supplement: Supplementary file 1 [file Supplementary_file_1.docx]

* SPSS SYNTAX FOR PRELIMINARY ANALYSES

* This syntax:

* 1) Opens the raw item-level data file (Raw_Data.sav).

* 2) Handles missing data via Expectation-Maximization (EM).

* 3) Computes scale scores for each construct.

* 4) Generates descriptive statistics & correlations.

* 5) Saves an imputed dataset for AMOS/SEM.

************************************************************************.

* STEP 0: OPEN THE RAW DATA (ASSUME .sav FORMAT).

GET FILE='C:\Path\To\ Raw_Data.sav'.

DATASET NAME RawData WINDOW=FRONT.

* STEP 1: DATA SCREENING FOR MISSING VALUES (EM).

MVA VARIABLES=CPCQ1 TO CPCQ40 MSSS1 TO MSSS12 GM1 TO GM8 AB1 TO AB4

/EM (TOLERANCE=0.001 CONVERGE=0.0001 ITERATE=25).

EXECUTE.

* Save the dataset with EM-imputed values.

SAVE OUTFILE='C:\Path\To\Imputed_Data.sav' /COMPRESSED.

DATASET ACTIVATE RawData.

* Re-open the newly saved imputed data file.

GET FILE='C:\Path\To\Imputed_Data.sav'.

DATASET NAME ImputedData.

* STEP 2: COMPUTE SCALE-LEVEL SCORES.

COMPUTE PsyCap = MEAN(CPCQ1 TO CPCQ40).

COMPUTE SocialSupport = MEAN(MSSS1 TO MSSS12).

COMPUTE GrowthMindset = MEAN(GM1 TO GM8).

COMPUTE AcademicBuoyancy = MEAN(AB1 TO AB4).

EXECUTE.

* STEP 3: DESCRIPTIVE STATISTICS & CORRELATIONS.

DESCRIPTIVES VARIABLES=PsyCap SocialSupport GrowthMindset AcademicBuoyancy

/STATISTICS=MEAN STDDEV.

CORRELATIONS /VARIABLES=PsyCap SocialSupport GrowthMindset AcademicBuoyancy

/PRINT=TWOTAIL NOSIG.

* Check skewness/kurtosis for normality assumptions.

FREQUENCIES VARIABLES=PsyCap SocialSupport GrowthMindset AcademicBuoyancy

/STATISTICS=SKEWNESS KURTOSIS.

* STEP 4: SAVE DATA FOR SEM IN AMOS.

SAVE OUTFILE='C:\Path\To\Ready_For_AMOS.sav' /COMPRESSED.

EXECUTE.
