# Supplementary material for: Intrapersonal strengths and interpersonal support: predicting academic buoyancy through psychological capital and growth mindset
Source: Front Psychol. 2025 Jul 17;16:1584343. doi: 10.3389/fpsyg.2025.1584343 (PMC12311807; doi:10.3389/fpsyg.2025.1584343)
Supplement: Supplementary file 2 [file Supplementary_file_2.docx]

**2. SPSS AMOS Script (Using Python Plugin)**

************************************************************************

* AMOS SCRIPT (PYTHON PLUGIN) EXAMPLE

************************************************************************.

BEGIN PROGRAM Python.

import spss, subprocess, os

# File paths: adjust to your local directories.

input_data = r"C:\Path\To\Ready_For_AMOS.sav"

output_amos_file = r"C:\Path\To\AMOS_ModelOutput.amw"

amos_script_file = r"C:\Path\To\AMOS_Script.aps"

# Construct the AMOS .aps script text:

# The example below assumes item-level modeling for:

# - PsyCap by CPCQ1-CPCQ40

# - SocialSupport by MSSS1-MSSS12

# - GrowthMindset by GM1-GM8

# - AcadBuoy by AB1-AB4

# Then structural paths: PsyCap -> AcadBuoy, SocialSupport -> AcadBuoy,

# PsyCap -> GrowthMindset, SocialSupport -> GrowthMindset,

# GrowthMindset -> AcadBuoy, plus covariances for exogenous factors.

script_text = f"""

FileName '{output_amos_file}';

DataFile '{input_data}';

/* Declare observed variables */

VariableNames

CPCQ1 CPCQ2 ... CPCQ40

MSSS1 MSSS2 ... MSSS12

GM1 GM2 ... GM8

AB1 AB2 AB3 AB4

;

/* Analysis properties: ML estimation, 5000 bootstrap samples */

AnalysisProperties

Estimation=ML

BootstrapSamples=5000

OutputBootstrapStandardizedEstimates=Yes

OutputStandardizedResiduals=Yes

;

/* Measurement model specification */

PsyCap by

CPCQ1, CPCQ2, ..., CPCQ40;

SocialSupport by

MSSS1, MSSS2, ..., MSSS12;

GrowthMindset by

GM1, GM2, ..., GM8;

AcadBuoy by

AB1, AB2, AB3, AB4;

/* Structural paths */

AcadBuoy on PsyCap SocialSupport GrowthMindset;

GrowthMindset on PsyCap SocialSupport;

/* Covariances among exogenous factors */

PsyCap with SocialSupport;

/* Request additional output */

Output

StandardizedRegressionWeights

SquaredMultipleCorrelations

Covariances

ResidualMoments

ModificationIndices(ParChange)

;

/* Run the calculations and save. */

Calculate;

SaveAs '{output_amos_file}';

"""

# Save the script to a .aps file

with open(amos_script_file, "w", encoding="utf-8") as f:

f.write(script_text)

# Now call the AMOS executable in batch mode (path may differ by version).

amos_exe = r"C:\Program Files\IBM\SPSS\Amos\27\AmosStarter.exe"

cmd = f'"{amos_exe}" "{amos_script_file}"'

subprocess.call(cmd, shell=True)

print("AMOS run complete. The .amw file and output can be found at:", output_amos_file)

END PROGRAM.
